# Face morphing attack detection based on high-frequency features and progressive enhancement learning

**DOI:** 10.3389/fnbot.2023.1182375

**Published:** 2023-06-05

**Authors:** Cheng-kun Jia, Yong-chao Liu, Ya-ling Chen

**Affiliations:** School of Electrical and Information Engineering, Hunan Institute of Traffic Engineering, Hengyang, China

**Keywords:** face morphing attacks, machine learning, high-frequency features, progressive enhancement learning, self-enhancement module, interactive-enhancement module

## Abstract

Face morphing attacks have become increasingly complex, and existing methods exhibit certain limitations in capturing fine-grained texture and detail changes. To overcome these limitation, in this study, a detection method based on high-frequency features and progressive enhancement learning was proposed. Specifically, in this method, first, high-frequency information are extracted from the three color channels of the image to accurately capture the details and texture changes. Next, a progressive enhancement learning framework was designed to fuse high-frequency information with RGB information. This framework includes self-enhancement and interactive-enhancement modules that progressively enhance features to capture subtle morphing traces. Experiments conducted on the standard database and compared with nine classical technologies revealed that the proposed approach achieved excellent performance.

## 1. Introduction

Facial features are widely used as personal identity authentication information. With the improvement in the recognition rate, face recognition systems are increasingly being used in bank businesses, mobile phone national ID card systems, face payment, and border management.

However, studies have revealed that face recognition systems are vulnerable to face morphing attacks (Scherhag et al., 2017) in which two facial images with various biological characteristics are synthesized into a morphed facial image with biometric information that is similar to the two facial images. A morphed face image results in face recognition systems matching two people. If such images are embedded in passports or other electronic travel documents, then border management systems can become vulnerable.

In many countries, applicants provide facial images for use in e-passport applications. Criminals can use free software to transform their facial images into those of friends with similar appearance. Because morphed faces are similar to real faces, if a partner uses the morphed face to apply for electronic travel documents, then criminals can use facial images on electronic travel documents to deceive border inspectors and recognition systems for passing automatic border control. Because such attacks have been proven to be effective (Ferrara et al., 2014), detecting faces generated by this attack is critical for social security.

Detection approaches are classified into conventional and depth-feature-based methods. Conventional feature-based methods include texture (Raghavendra et al., 2016, 2017; Venkatesh et al., 2020) and quality-based methods (Makrushin et al., 2017; Debiasi et al., 2018a,b; Scherhag et al., 2019). With deep learning technology evolving rapidly, the method based on depth feature (Seibold et al., 2017; Long et al., 2022, 2023) is widely used. Among these methods, conventional feature methods are simple to implement but cannot achieve satisfactory discriminability. By contrast, although depth-feature-based methods can extract semantic information effectively and exhibit superior generalization, these methods tend to extract global information from images and ignore details. Studies (Luo et al., 2021) have revealed that existing deep learning methods exhibit poor performance in recognizing realistic synthetic faces because they cannot extract details effectively.

With advancement in morphing attack technology (Makrushin et al., 2017; Qin et al., 2020), morphed faces are becoming increasingly realistic, rendering discerning the differences between real and morphed images due to subtle and localized differences difficult. Consequently, the limitations of existing methods are especially concerning. To address this problem, a novel face morphing attack detection method based on high-frequency features and progressive enhancement learning was proposed to effectively extract details and overcome the limitations of existing methods. The contributions of this study are as follows:

- A novel face morphing detection method based on high-frequency features was proposed. High-frequency features typically represent parts of the image with high variation rates, including details and texture information. The use of high-frequency information as the input to a neural network can better capture image details, thereby improving the performance and accuracy of the model in detecting morphed images.
- A progressive enhancement learning framework based on two-stream networks was proposed for training a detection model. The framework comprises of self-enhancement and interactive-enhancement modules. These modules gradually improve the feature representation of the model, and enable it to accurately capture subtle morphing traces.
- The proposed system is analyzed on the standard database. Experiments on two databases revealed excellent performance in the single- and cross-dataset tests.

The rest of the paper is organized as follows: Section 2 introduces the related work. Section 3 depicts the proposed method. Section 4 provides experimental results and analysis. Finally, Section 5 presents conclusions.

## 2. Related work

Face morphing detection is a critical task for ensuring social security. Various techniques have been proposed to address this problem. In this section, we review several state-of-the-art methods for detecting face morphing. Specifically, we categorized these methods into three types, namely texture-based methods, image-quality-based methods, and depth-feature-based methods. We discussed the strengths and weaknesses of each method and highlighted the necessity of effective and accurate techniques to detect face morphing.

### 2.1. Face morphing detection based on texture

Raghavindra et al. (2016) proposed the use of binary statistical image features (BSIF) to detect morphed faces. The method was tested on a large database consisting of 450 morphed face images created by 110 subjects of different races, ages, and genders. Experimental results proved that the method is efficient. Subsequently, Raja et al. proposed a method by using multi-color spatial features (Raghavendra et al., 2017). In this method, texture features extracted from HSV and YCbCr were used for detection. The bona fide presentation classification error rate (BPCER) of this method was 1.73%, and the attack presentation classification error rate (APCER) was 7.59%, which revealed superior detection performance compared to earlier methods. Venkatesh et al. proposed the use of multiple features to improve detection performance (Venkatesh et al., 2020). In this method, BSIF, HOG, and LBP were used to extract features. Compared with earlier studies, this model exhibited stable detection performance under various environments and conditions.

### 2.2. Face morphing detection based on image quality

Neubert et al. proposed an automated detection approach based on JPEG degradation of continuous images (Makrushin et al., 2017). Under laboratory conditions, the accuracy rate was 90.1%, and under real world conditions, the accuracy rate was 84.3%. Photo response non-uniformity (PRNU) is a source of mode noise in digital cameras and is generated when photons in a digital image sensor are converted into electrons. PRNU features are widely used in image forgery detection because operations such as image copying or moving changes the PRNU features of images. Therefore, Debiasi et al. (2018a) proposed the use of PRNU features for detection. According to experimental results, PRNU analysis achieved reliable detection performance for morphed faces and maintained excellent performance even under image scaling and sharpening. Debiasi et al. (2018b) proposed an improved version of the PRNU. Two detection methods based on the PRNU were used to analyze the Fourier spectrum of PRNU and statistical methods were used for quantifying the spectral distinction between real and morphed face images. The value of PRNU was affected by the fusion operation in both spatial domain and frequency domains. Scherhag et al. (2019) introduced spatial features for the parallel analysis of frequency domain features.

### 2.3. Face morphing detection based on depth feature

In most morphing detection methods, deep learning methods, especially the pre-trained CNN architecture, is used. Seibold et al. (2017) first proposed a detection approach on the basis of deep learning. Three popular network structures, namely AlexNet, GoogLeNet, and VGG19 were evaluated. Experimental results revealed that VGG19 after pre-processing can obtain excellent performance. In subsequent studies, evaluation has been gradually combined with ResNet, Inception, and other networks. Subsequently, Long et al. used the lightweight network structure and local feature information to improve accuracy. The network achieved high accuracy with fewer parameters (Long et al., 2022). To enhance the generalization ability of the network, Long et al. (2023) proposed a detection method based on a two-stream network with the channel attention mechanism and residual of multiple color spaces. In the method, the residual noise of multiple space and attention mechanism were used to detect morphed face. Experimental results revealed that the proposed method outperformed existing methods.

Methods based on conventional features are simple to implement but cannot achieve satisfactory discriminability, whereas methods based on deep feature generally outperform conventional methods but tend to extract global information from images and ignore details. To overcome the limitations of existing methods, a detection method based on high-frequency features and progressive enhancement learning was proposed for detecting morphed faces. High-frequency features typically represent parts of the image with high variation rates, including details and texture information. The use of high-frequency information as the input to a neural network can enhance the details of the captured image. Progressive enhancement learning is a learning method that progressively enhances feature representations. It achieves this by inserting self-enhancement modules after each convolution block in a convolutional neural network, and interactive-enhancement modules after each stage to gradually enhance the feature representation. This method effectively utilizes high-frequency information to better locate subtle morphing traces.

## 3. Proposed method

**Figure 1:**
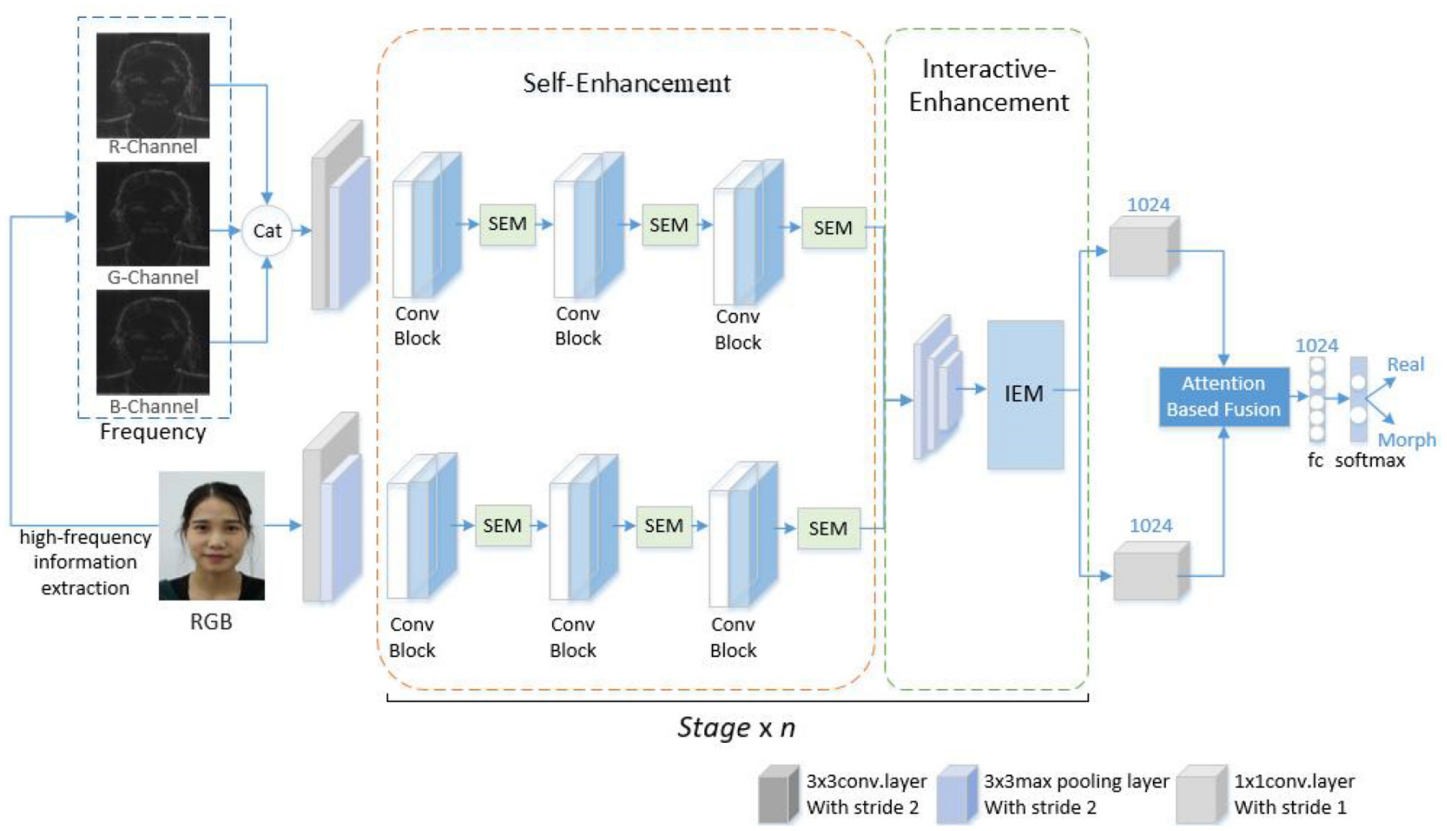
Presented approach.

The proposed scheme is displayed in Figure 1. The scheme can detect the morphed face image by using high-frequency features and progressive enhancement learning. First, the image is preprocessed and subsequently decomposed into R, G, B color channels. High-frequency features are extracted from images in R, G, B channels. Finally, the merged high-frequency information image and RGB image are input into the designed progressive enhancement learning framework for end-to-end training for detecting morphed faces. The scheme consists of three parts, namely pre-processing, high-frequency information extraction, and progressive enhancement learning framework design. Each part is described in this paper.

### 3.1. Pre-processing

To effectively extract features from the image, pre-processing the image is critical. In the pre-processing stage, first, the dlib detector was used for face detection (King, 2009). The detected faces were then cropped to 224 × 224 pixels to ensure the morphing detection algorithm was applied to the face area. Next, 224 × 24 pixels were selected to accommodate the size of the input layer of the progressive enhanced two-stream network.

### 3.2. High-frequency information extraction

High-frequency information contains considerable detail information as well as noise. Detailed information can be used to detect subtle differences between real and the morphed faces, and noise can suppress the image content. Therefore, high-frequency information was introduced to detect morphed faces.

To extract the high-frequency information of facial image *X*, the high-frequency information of R, G, B color channels was extracted. First, the input image was decomposed into R, G, and B three channels, and the separated images were represented as *X*_*r*_, *X*_*g*_, and *X*_*b*_. The corresponding frequency spectra *X*_*fr*_, *X*_*fg*_, and *X*_*fb*_ are obtained through Fourier transform as follows:


(1) $$\begin{array}{l} RGB\left(X\right)=\left[X_{r}\;,\;X_{g}\;,\;X_{b}\right]\text{,} \end{array}$$

(2) $$\begin{array}{l} X_{fr},X_{fg},X_{fb}=D\left(X_{r},X_{g},X_{b}\right)\text{,} \end{array}$$

Where, $X_{fr}\text{,X}f_{g}\text{,X}f_{b}\in R^{H\times W\times 1}$, and *D* represents the discrete Fourier transform (DFT). The image obtained after DFT transformation exhibits excellent frequency distribution layout, that is, the low-frequency response is at the top corner and high-frequency response is at the lower right corner. To extract high-frequency information, the low-frequency part of the upper left corner is moved to the middle. The specific operation symmetrically exchanges the four quadrants of the frequency domain image, that is, the first and third quadrants, the second and fourth quadrants exchange positions. Thus, the zero-frequency component is moved to the center of the spectrum. Next, the image content is suppressed by filtering low-frequency information for magnifying high-frequency subtle artifacts as follows:


(3) $$\begin{array}{l} X_{f_{r}}^{h},X_{f_{g}}^{h},X_{f_{b}}^{h}=F(X_{fr},a),F(X_{fg},a),F(X_{fb},a), \end{array}$$

Where, *F* represents high-pass filtering, α controls the low-frequency components to be filtered. Generally, the value range of α is limited between [0.1, 0.5] because within this range, the value of α can not only filter low-frequency components to a certain extent but also retain the high-frequency information in the image to achieve superior filtering effect. Therefore, the value of α was set to 0.33. Finally, the frequency spectrum with high-frequency information was converted into RGB color space by using inverse Fourier transform to obtain the output image with high-frequency information as follows:


(4) $$\begin{array}{l} X_{hr},X_{hg},X_{hb}=D^{-1}(X_{fr}^{h},X_{fg}^{h},X_{f_{b}}^{h})\text{ ,} \end{array}$$

Where, $X_{hr}{\text{,X}}_{hg}{\text{,X}}_{hb}\in R^{H\times W\times 1}$, and *D*^−1^ represents inverse discrete Fourier transform (IDFT). Finally, the high-frequency information images extracted from the three channels are spliced along the channel direction to obtain the final high-frequency feature image as follows:


(5) $$\begin{array}{l} X^{h}=cat\left(X_{hr},X_{hg},X_{hb}\right)\text{,} \end{array}$$

**Figure 2:**
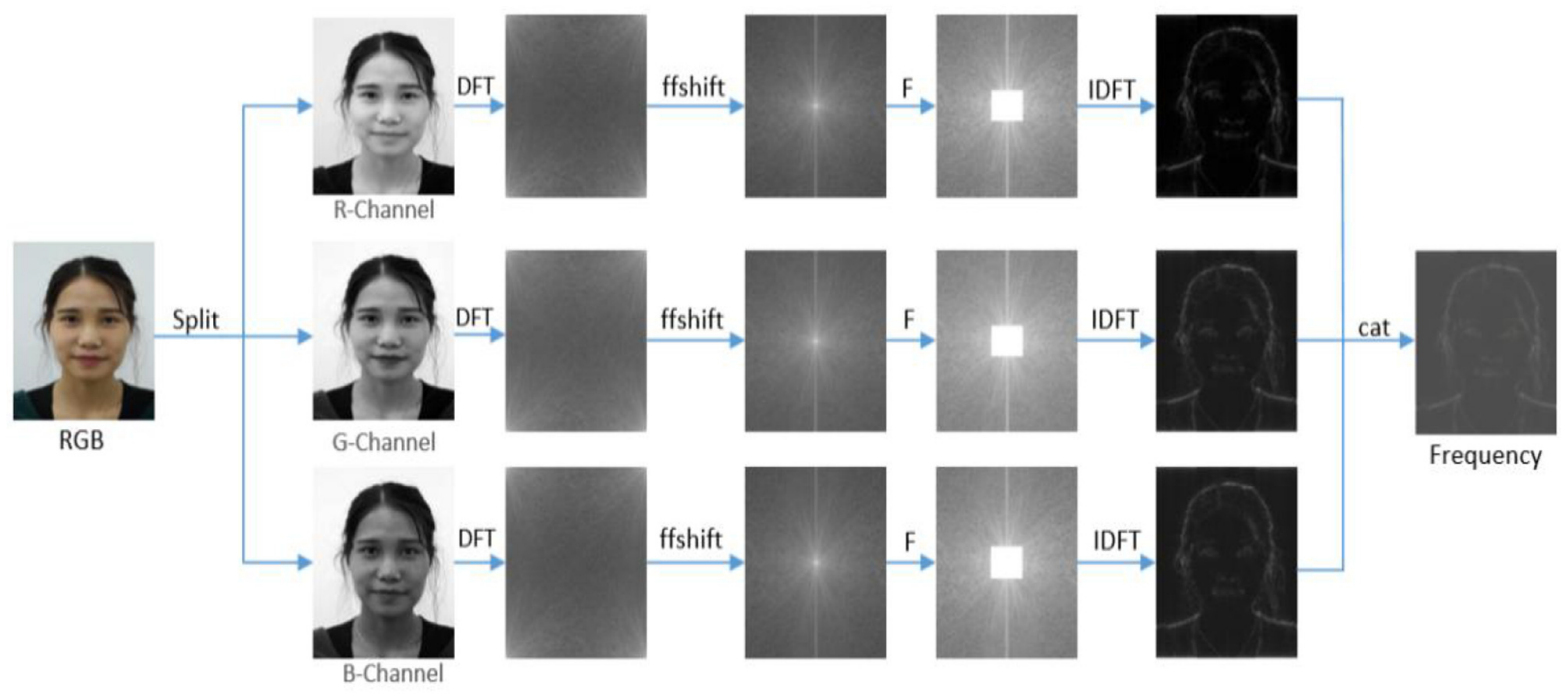
Extraction process of three-channel high-frequency features.

The high-frequency information extraction process is displayed in Figure 2.

### 3.3. Progressive enhancement learning framework

Attention mechanisms are widely used in image processing tasks (Sa et al., 2021; Gu et al., 2022). Inspired by these mechanisms, this study proposed a progressive enhancement learning framework (PELF) to enhance detection performance by combining RGB image information with high-frequency information. The RGB image information provides basic color and shape information, whereas the high-frequency image information provides detailed information. Fusing RGB and high-frequency features enables a comprehensive feature representation, which results in improved detection performance.

**Figure 3:**
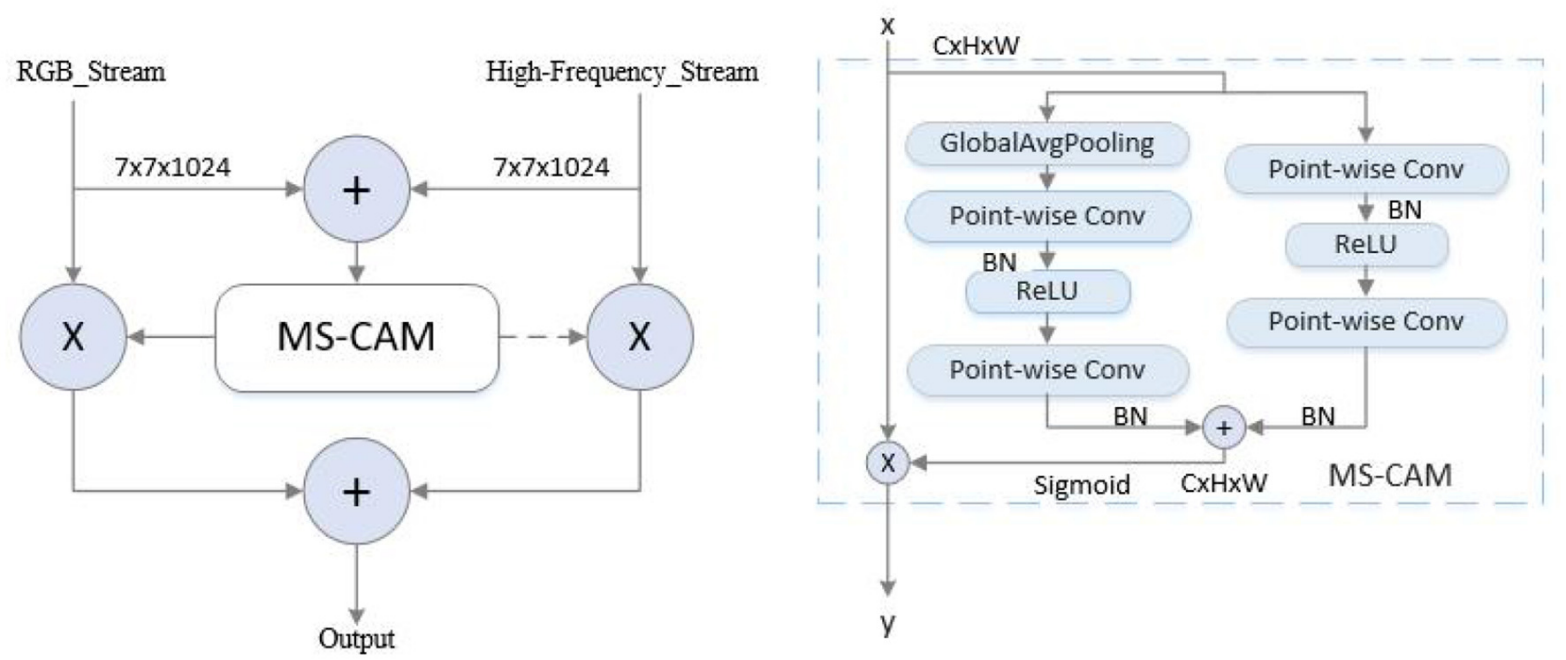
AFF module.

The framework is based on a two-stream network architecture, where RGB images and corresponding high-frequency information images are simultaneously fed into the network as the input. The backbone network is ShuffleNetV2, which is end-to-end trained. To enhance the features in both intra- and inter-stream manner, self-enhancement modules and interactive-enhancement modules are designed. Specifically, each convolutional block of the backbone is followed by a self-enhancement module, and interactive-enhancement modules are inserted after each stage. The self-enhancement module can enhance the characteristics of each flow. The interactive-enhancement module can enhance the feature interaction between RGB and high-frequency information. This progressive feature enhancement process effectively locates subtle morphing traces and improves detection performance. In the feature fusion stage, the AFF module (Dai et al., 2021) is used to fuse RGB and high-frequency features, as displayed in Figure 3. This method is a feature fusion method, which can use the complementarity and correlation between the two features and improve the expression ability and classification performance of features. After passing through the AFF module, the output dimension remains consistent with the input dimension, which is 7 × 7 × 1024. The resulting fused features are then sent to the Softmax layer for classification.

#### 3.3.1. Self-enhancement module

**Figure 4:**
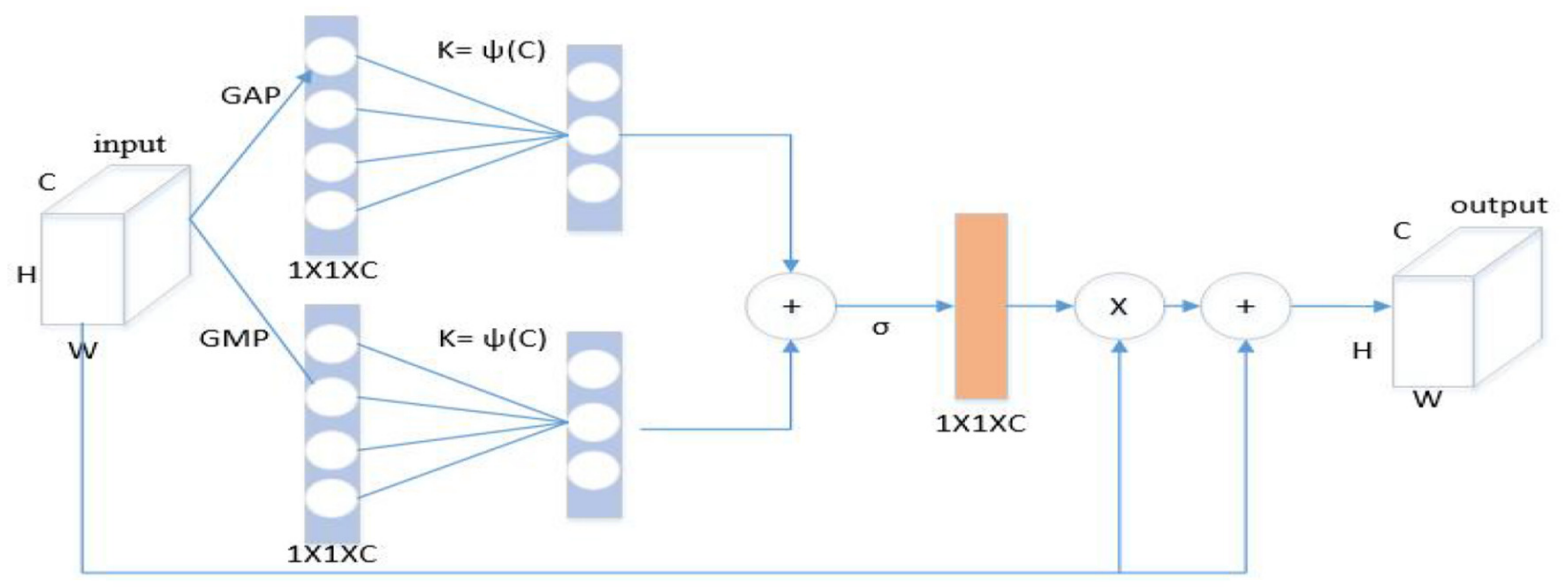
Self-enhancement module.

Inspired by the channel attention mechanism, a self-enhancement module (Figure 4) was designed to enhance the characteristics of each flow. Specifically, the global features of each channel were extracted through global average pooling (GAP) and global max pooling (GMP), and the global spatial features of each channel was considered as the representation of the channel to form a 1 × 1 × *C* channel descriptor. The description is as follows:


(6) $$\begin{array}{l} S\text{1}=GAP\left(f_{in}\right)\text{, S2}=GMP\left(f_{in}\right)\text{,} \end{array}$$

Where, *f*_*in*_ represents the input feature map. To effectively capture cross-channel interaction information, this paper considers capturing local cross-channel interaction information from each channel and its *k* neighbors. For this purpose, we subject the obtained global spatial features *S1* and *S2* to fast one-dimensional convolution with a kernel size of *k*. These operations generate two channel attention maps, *Z1* and *Z2*, which are obtained by passing the convolved features through a sigmoid function. The description is as follows:


(7) $$\begin{array}{l} Z\text{1}=\sigma \left(C1D_{k}\left(S\text{1}\right)\right)\text{,}Z\text{2}=\sigma \left(C1D_{k}\left(S\text{2}\right)\right)\text{,} \end{array}$$

Where, *C1D* represents one-dimensional convolution, σ represents Sigmoid function, and convolution kernel size *k* represents the number of neighbors participating in attention prediction near this channel. Here, the final channel attention map *Z* is computed by adding *Z1* and *Z2* together. This map is then used to multiply the input characteristics of each flow *f*_*in*_, leading to an enhanced feature representation. Finally, the enhanced feature is added to the original input feature, resulting in the final output *f*_*out*_. The description is as follows:


(8) $$\begin{array}{l} Z=Z1+Z2\text{,} \end{array}$$

(9) $$\begin{array}{l} f_{out}=f_{in}+f_{in}⊗Z\text{,} \end{array}$$

Where, *f*_*out*_ represents the output feature after passing through the module. The self-enhancement module was inserted after each convolution block. Through channel attention, the trajectories in various input spaces were captured to enhance the characteristics of each flow.

#### 3.3.2. Interactive-enhancement module

**Figure 5:**
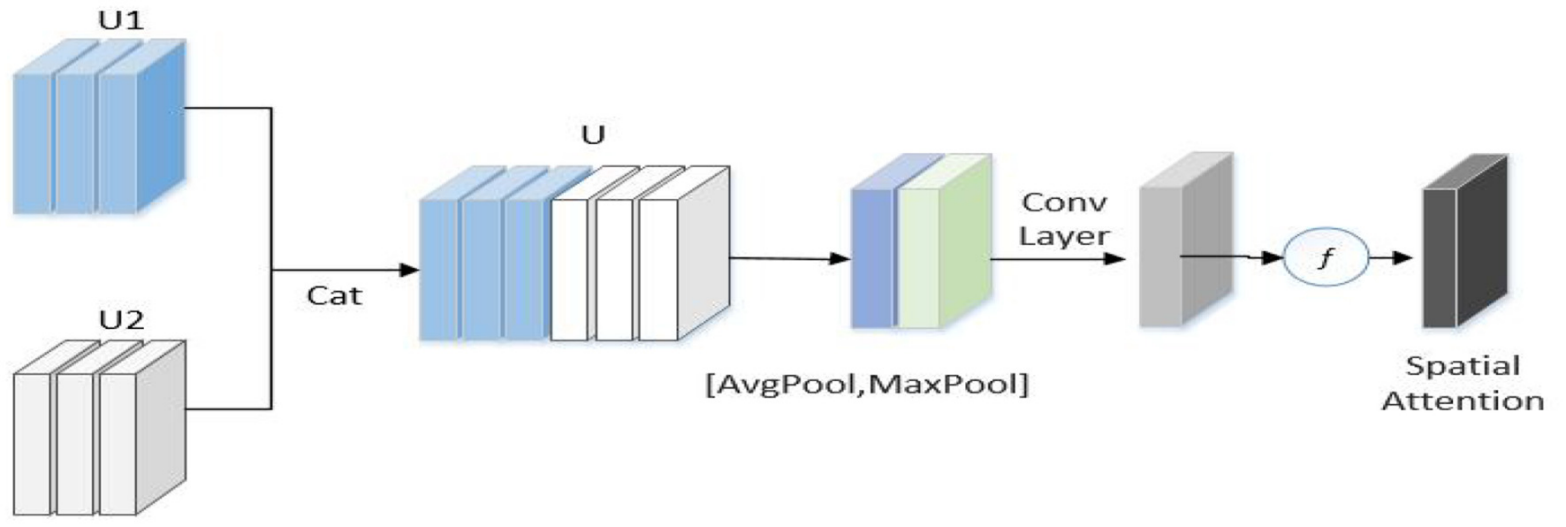
Interactive-enhancement module.

To exploit RGB information and high-frequency information, an interactive-enhancement module (Figure 5) was used to enhance the interaction of two-stream features.

As displayed in Figure 5, *U1* and *U2* represent the feature map of the frequency flow and RGB flow, respectively, of the *l*-th stage of the network, and H, W, and C represent the length, width, and height, respectively, of the feature map. First, U1 and U2 are connected in the channel dimension to obtain *U*. Next, *U* is used to generate effective feature descriptors through GAP and GMP operations, and a 7 × 7 convolution operation was performed to reduce the dimension to one channel. The spatial attention feature is generated by sigmoid. Finally, this feature is multiplied with the input feature of each flow to obtain the enhanced feature as follows:


(10) $$\begin{array}{l} \begin{array}{lll} V & = & \sigma (f^{7\times 7}([AvgPool(U);MaxPool(U)]))=\sigma (f^{7\times 7}(Z_{avg}^{s};Z_{\max}^{s}))\; \end{array} \end{array}$$

(11) $$\begin{array}{lll} Uz & = & V⊗U\text{i ,} \end{array}$$

Where, ⊗ represents element multiplication, σ represents the sigmoid function, and *U*_*Z*_ is the feature of each stream enhanced by the interactive-enhancement module. The module is inserted after each stage and placed after the self-enhancement module. Thus, the enhancement of RGB and high-frequency branches can be realized simultaneously.

## 4. Experimental results and analysis

### 4.1. Datasets and evaluation criteria

FEI and HNU datasets (Zhang L. B. et al., 2018; Peng et al., 2019) were used, and splicing the morphing attack was the primary attack mode. The images in the HNU dataset were collected from Chinese people and cover the face data of various genders. To ensure an excellent fusion effect, the individuals of the same age were selected, and the same lighting and background conditions were used. When evaluating the effectiveness of face fusion, four sub-protocols were included in the HNU for evaluating generalization. The pixel fusion factor corresponding to the four sub-protocols differs from the location fusion factor. In HNU (MDB1), the pixel fusion factor and position fusion factor are both fixed at 0.5, which reveals that the two faces as fusion materials that exhibit the same contribution to the fusion photograph. In this scenario, the attack effect is the best scenario because in this case, the fusion face image exhibits considerable similarity to the holder from the perspective of vision or face recognition systems. In practice, fusion photographs may be fused in various proportions of pixels and positions. To simulate the real scenario, the pixel fusion factor and position fusion factor of HNU (MDB2) and HNU (MDB3) were randomly selected with values ranging from 0.1 to 0.9, respectively. In HNU (MDB4), both factors were randomly selected. In the FEI dataset, Europeans and Americans are collection objects. In this dataset, both position fusion factor and pixel fusion factor are fixed values of 0.5. The details of the two datasets are presented in Table 1.

**Table 1:** HNU and FEI dataset.

**Dataset**	**Training set**		**Validation set**		**Testing set**		**Pixel fusion factor**	**Position fusion factor**
	**Real face**	**Morphed face**	**Real face**	**Morphed face**	**Real face**	**Morphed face**		
HNU (MDB1)	1,121	1,121	564	330	566	377	0.5	0.5
HNU (MDB2)	1,121	1,125	564	567	566	567	0.5	0.1–0.9
HNU (MDB3)	1,121	1,125	564	567	566	567	0.1–0.9	0.5
HNU (MDB4)	1,121	1,134	564	567	566	567	0.1–0.9	0.1–0.9
FEI	81	6,480	20	380	99	9,702	0.5	0.5

To assess the effectiveness of proposed scheme, the experimental results of this method were compared with nine existing classical methods. The results are presented in Tables 2, 3. In the case of deep learning technology, the results of the method were compared with VGG16 (Seibold et al., 2017), PLFL (Long et al., 2022), TSCR (Long et al., 2023), ResNet34 (He et al., 2016), ShuffleNet (Zhang X. et al., 2018), MobileNet (Sandler et al., 2018). In the case of non-deep learning technology, the method was compared with BSIF (Raghavendra et al., 2016), FS-SPN (Zhang L. B. et al., 2018), and HOG (Scherhag et al., 2018).

**Table 2:** Experimental settings.

**Parameter**	**Value**
Framework	Pytorch
Optimizer	Stochastic gradient descent (SGD)
Learning rate	1e-4
Loss criterion	Cross-entropy loss
Epochs	20
GPU	GeForce GTX 1060Ti
Batch size	16

**Table 3:** Detection results of the presented approach on fixed fusion factor datasets.

**Algorithm**	**FEI**			**HNU (MDB1)**		
	**EER (%)**	**BPCER@APCER**		**EER (%)**	**BPCER@APCER**	
		=**5%**	=**10%**		=**5%**	=**10%**
**Traditional technologies**						
BSIF-SVM (Raghavendra et al., 2016)	3.38	8.67	4.79	20.80	22.60	19.82
HOG-SVM (Scherhag et al., 2018)	3.03	0.40	0.60	24.84	62.90	48.39
FS-SPN (Zhang L. B. et al., 2018)	0.51	1.58	0.35	1.93	1.53	1.21
**Deep learning technologies**						
VGG16 (Seibold et al., 2017)	2.93	2.55	2.01	11.06	15.33	13.95
ResNet34 (He et al., 2016)	3.95	2.01	1.33	3.00	4.45	2.53
ShuffleNetV2 (Zhang X. et al., 2018)	2.02	2.02	1.01	4.01	3.98	1.89
MobileNetV2 (Sandler et al., 2018)	3.95	1.01	1.01	3.53	3.36	1.41
PLFL Long et al., 2022	0.85	0.98	0.55	0.91	1.35	0.37
TSCR (Long et al., 2023)	1.04	1.09	0.66	0.88	1.31	0.37
**Proposed method**	**0.12**	**0.66**	**0.17**	**0.84**	**0.36**	**0.18**

The bold values represent the best results.

Furthermore, standardized ISO metrics (Biometrics, 2016): APCER, BPCER, ACER, ACC and EER were used to evaluate detection performance. Here, APCER defines the proportion of the morphed image that is incorrectly classified as the real image, BPCER defines the proportion of real image that is incorrectly classified as the morphed image, ACER is defined as the average of BPCER and APCER. Furthermore, the results of EER, where BPCER = APCER, were provided.

### 4.2. Implementation details

The proposed approach is based on the Pytorch deep learning framework. In the training stage, the stochastic gradient descent (SGD) optimizer was used to optimize two branches, with a learning rate set to 1e-4. The loss criterion used was cross-entropy loss. The two branches were trained for 20 epochs on a GeForce GTX 1060Ti GPU, with a batch size value of 16. A summary table listing the parameters and criteria used for all algorithms is presented in Table 2 for easy comparison.

### 4.3. Experimental results and analysis

#### 4.3.1. Single-dataset experiment and analysis

In a single-dataset comparison experiment, the proposed method was compared with the conventional method and the deep learning-based method for verifying the effectiveness of the method. Table 3 indicates the quantitative results of the presented approach with nine classical approaches.

The proposed approach indicates that the performance of the EER was 0.12% with BPCER = 0.66% @APCER=5%, and BPCER = 0.17% @APCER= 10% on FEI. On HNU (MDB1), the EER is 0.84% with BPCER = 0.36% @APCER = 5%, and BPCER = 0.18% @APCER = 10%. Excellent results were obtained on both FEI and HNU (MDB1) datasets. The accuracy of the conventional method is low, and it exhibits considerable limitations as the feature extraction method. However, the effect of deep learning is superior to that of conventional methods, which indicates that deep learning technology exhibits obvious advantages. The performance of the proposed approach was verified on datasets with various pixel fusion factors, and Table 4 indicates relevant results.

**Table 4:** Results of the presented approach on various fusion factors datasets.

**Algorithm**	**HNU (MDB2)**			**HNU (MDB3)**			**HNU (MDB4)**		
	**EER (%)**	**BPCER@ APCER**		**EER (%)**	**BPCER@ APCER**		**EER (%)**	**BPCER@ APCER**	
		=**5%**	=**10%**		=**5%**	=**10%**		=**5%**	=**10%**
**Traditional technologies**									
BSIF-SVM (Raghavendra et al., 2016)	20.39	20.27	18.17	19.38	21.67	17.79	21.36	23.67	18.08
HOG-SVM (Scherhag et al., 2018)	22.72	61.02	46.91	21.16	47.09	31.92	23.46	59.61	48.50
FS-SPN (Zhang L. B. et al., 2018)	1.56	2.02	1.01	1.49	1.01	0.49	1.69	1.41	0.18
**Deep learning technologies**									
VGG16 (Seibold et al., 2017)	12.05	18.44	10.88	17.78	15.43	12.02	14.45	11.56	9.22
ResNet34 (He et al., 2016)	3.52	1.33	0.55	3.44	1.51	0.37	4.53	2.72	0.88
ShuffleNetV2 (Zhang L. B. et al., 2018)	4.06	3.53	1.41	9.19	14.66	8.66	7.60	14.49	4.42
MobileNeV2 (Sandler et al., 2018)	4.59	4.59	2.65	5.65	6.18	2.83	4.94	4.77	2.30
PLFL (Long et al., 2022)	1.00	0.37	**0.12**	1.24	1.21	**0.31**	1.21	1.51	0.18
TSCR (Long et al., 2023)	0.98	0.59	**0.12**	1.21	1.01	0.57	1.16	1.41	0.16
Proposed method	**0.88**	**0.27**	**0.12**	**1.06**	**0.65**	0.35	**0.77**	**0.37**	**0.05**

The bold values represent the best results.

For the presented approach, the EER was 0.88% on HNU (FaceMDB2), the EER was 1.06% on HNU (FaceMDB3), and the EER was 0.77% on HNU (FaceMDB3). Compared with nine MAD technologies, the proposed approach achieved excellent detection results on datasets with various pixel fusion factors. Under various pixel fusion and position fusion factors, the proposed approach was still robust.

#### 4.3.2. Cross-dataset experiments and analysis

The cross-dataset test was conducted for verifying the generalization ability of the approach. HNU (MDB1) and FEI datasets were used in the study. The common feature of these two datasets is that the position fusion factor and pixel fusion factor were fixed at 0.5. Table 5 indicates relevant results.

**Table 5:** Detection results on cross dataset.

**Training dateset**	**Test dateset**	**Algorithms**	**EER (%)**	**BPCER@APCER**	
				=**5%**	=**10%**
HNU (MDB1)	FEI	Traditional technologies			
		BSIF-SVM (Raghavendra et al., 2016)	30.27	87.77	63.16
		HOG-SVM (Scherhag et al., 2018)	40.01	60.01	50.33
		FS-SPN (Zhang X. et al., 2018)	37.37	85.69	75.02
		Deep learning technologies			
		VGG16 (Seibold et al., 2017)	10.22	12.16	10.24
		ResNet34 (He et al., 2016)	5.65	8.08	4.55
		ShuffleNet (Zhang L. B. et al., 2018)	12.63	24.24	15.15
		MobileNet (Zhang L. B. et al., 2018)	15.87	35.35	23.23
		PLFL (Long et al., 2022)	4.52	3.30	1.51
		TSCR (Long et al., 2023)	4.48	**2.02**	**1.01**
		**Proposed method**	**3.26**	3.03	1.47
FEI	HNU (MDB1)	Traditional technologies			
		BSIF-SVM (Raghavendra et al., 2016)	40.09	81.86	47.19
		HOG-SVM (Scherhag et al., 2018)	35.48	87.10	80.97
		FS-SPN (Zhang X. et al., 2018)	25.09	60.07	45.09
		Deep learning technologies			
		VGG16 (Seibold et al., 2017)	10.53	20.26	10.22
		ResNet34 (He et al., 2016)	17.47	45.58	23.04
		ShuffleNet (Zhang L. B. et al., 2018)	16.08	39.40	22.08
		MobileNet (Sandler et al., 2018)	26.68	65.02	51.24
		PLFL (Long et al., 2022)	8.33	**11.25**	5.84
		TSCR (Long et al., 2023)	**7.95**	12.54	**4.77**
		**Proposed method**	10.22	14.25	5.25

The bold values represent the best results.

In the cross-dataset test, the overall effect was reduced compared with the single-dataset experiment because the various methods of obtaining images from different datasets or races of individuals as materials. When HNU (MDB1) was used as the training set and tested on FEI, the EER value of presented approach was 3.26%. By contrast, when using FEI as the training set and HNU (MDB1) test, the EER value of proposed approach was 10.22%. Furthermore, the proposed approach can achieve excellent generalization ability.

#### 4.3.3. Ablation experiment and analysis

(1) Ablation experiment for the two-stream network

Ablation experiments were conducted to verify the effectiveness of the designed two-stream convolution neural network. Table 6 indicates relevant results.

**Table 6:** Ablation results for the two-branch network.

	**FEI**			**HNU (MDB1)**		
	**ACER (%)**	**EER (%)**	**ACC (%)**	**ACER (%)**	**EER (%)**	**ACC (%)**
High- frequency-CNN	1.55	0.55	98.80	1.99	1.74	98.13
RGB-CNN	6.08	2.59	97.21	7.07	3.00	94.16
TSCNN	**0.67**	**0.32**	**98.93**	**1.95**	**0.88**	**98.26**

The bold values represent the best results.

The effect of the high-frequency stream is superior than the RGB stream under the same conditions. This phenomenon indicates that distinguishing between real and the morphed face in the RGB color space is difficult, whereas the high-frequency stream can directly identify the difference between two categories of images. On the FEI dataset, the ACER of the TSCNN was 0.67%, the EER was 0.32%, and the ACC was 98.93%. On the HNU (MDB1) dataset, ACER was 1.95%, EER was 0.88%, and ACC was 98.26%. On the two datasets, the performance of the two-branch network achieved performance superior to that of the single-branch network. This phenomenon indicates that the fusion of the two branches contributes to a comprehensive feature representation.

(2) The ablation experiment for self-enhancement module and interactive-enhancement module

To highlight the contribution of self-enhancement module (SEM) and interactive- enhancement module (IEM) to the detection system, an ablation study was conducted on two datasets, and the relevant results are presented in Table 7.

**Table 7:** Ablation results for two enhancement modules.

**Algorithm**	**FEI**			**HNU (MDB1)**		
	**ACER (%)**	**EER (%)**	**ACC (%)**	**ACER (%)**	**EER (%)**	**ACC (%)**
TSCNN	0.67	0.32	98.93	1.95	0.88	98.26
TSCNN+SEM	0.33	0.20	99.57	1.91	0.88	98.46
PELF (ours)	**0.08**	**0.12**	**99.83**	**1.59**	**0.84**	**98.70**

The bold values represent the best results.

After introducing the designed self-enhancement and interactive-enhancement modules, on the FEI dataset, the ACER was 0.08%, the EER was 0.12%, and the ACC was 99.83%. On the HNU (MDB1) dataset, ACER was 1.59%, EER was 0.84%, and ACC was 98.70%. The SEM enhanced the characteristics of each flow, whereas the interactive-enhancement module can complement each other to enhance the feature interaction of dual flows. Therefore, performance on both modules improved. Through this progressive feature enhancement process, high-frequency information and RGB information can be effectively used to subtle morphing traces.

## 5. Discussion

The findings of our experiments demonstrated the effectiveness of the proposed method in detecting morphing attacks. Specifically, we compared the proposed method with methods on both single and cross-dataset evaluations, and the results revealed that the method achieved lower equal error rate. These results are particularly significant given the increasing prevalence of morphing attacks in various security-sensitive applications.

Furthermore, ablation experiments on the dataset demonstrated the critical importance of incorporating high-frequency features and a progressive enhancement learning framework into the detection process. The use of high-frequency features and a progressive enhancement learning framework based on two-stream networks considerably improved the performance of the model. High-frequency features are crucial in distinguishing between morphed and authentic images, as they can capture subtle differences that may not be visible to the naked eye. Moreover, the progressive enhancement learning framework enables the model to learn more discriminative features.

## 6. Conclusion

Morphed face detection is critical for mitigating illegal activities. Based on the conventional deep learning binary classification, a novel detection framework based on high-frequency features and progressive enhanced two-branch network structure was proposed. RGB stream and high-frequency information stream were used to simultaneously detect morphed faces and enhance the feature interaction of two streams by using the SEM and IEM. The robustness and generalization of the approach were verified on HNU and FEI datasets. In the future, high-frequency features and a progressive enhanced two-stream network can be used for detecting differential morphing attacks.

## Data availability statement

The original contributions presented in the study are included in the article/supplementary material, further inquiries can be directed to the corresponding author.

## Ethics statement

Written informed consent was obtained from the individual(s) for the publication of any potentially identifiable images or data included in this article.

## Author contributions

Conceptualization and validation: C-kJ and Y-cL. Investigation and writing–review and editing: Y-cL. Writing–original draft preparation: C-kJ. Supervision: C-kJ and Y-lC. Formal analysis: Y-lC. All authors have read and agreed to the published version of the manuscript.
